# Feasibility and acceptability of a web-intervention to prevent alcohol and cannabis-impaired driving among adolescents in driver education

**DOI:** 10.21203/rs.3.rs-4249553/v1

**Published:** 2024-04-19

**Authors:** Katherine Nameth, Elizabeth Ueland, Elizabeth J. D’Amico, Karen Chan Osilla

**Affiliations:** Stanford University School of Medicine; Stanford University School of Medicine; RAND Corporation; Stanford University School of Medicine

**Keywords:** Adolescents, Impaired Driving, Alcohol, Cannabis, Risky driving behaviors, Web intervention, Prevention

## Abstract

**Background:**

Adolescents increasingly view cannabis as a substance with limited harm. Their propensity to engage in risky driving, combined with their relative driving inexperience, places adolescents at heightened risk for harm resulting from impaired driving. Driver education provides an opportunity to help prevent and reduce these risks, yet few interventions address cannabis-impaired driving, especially impairment from simultaneous use of both cannabis and alcohol.

**Methods:**

We adapted a single-session primary care brief intervention (CHAT) for driver education programs. First, we conducted two focus groups with adolescents aged 15–17 years (n = 6; n = 5) enrolled in driver education programs. Their feedback was integrated into a prototype of an online intervention called webCHAT that focuses on preventing alcohol and cannabis-impaired driving. Next, we recruited a new sample of adolescents who user tested webCHAT (n = 8) and provided qualitative and survey feedback. We analyzed qualitative data using classic content analysis and grouped themes according to the feasibility and acceptability of webCHAT.

**Results:**

Participants suggested that webCHAT should have adolescent narrators in short, informal, and interactive videos. In satisfaction surveys (n = 8), 88% of participants would recommend webCHAT to a friend and 88% reported that they learned helpful skills regarding impaired driving. General acceptability was also reflected in interviews (n = 6; 100% would recommend the intervention to a friend, 100% indicated overall positive impressions, and 67% stated it was easy to use). Participants reported that it was helpful to learn about the negative effects of both cannabis and alcohol on driving behavior, voicing that webCHAT would help adolescents make more informed decisions.

**Conclusions:**

Soliciting adolescent perspectives is critical when developing interventions targeting cannabis use because of increasingly permissive attitudes and perceptions of minimal risk associated with use. The current study highlights how feedback can help increase both the feasibility and acceptability of interventions.

## Introduction

Preventing risky driving behaviors in adolescents is a top priority for public health. Motor vehicle crashes are the leading cause of death in adolescents 15 to 20 years old [1]. Furthermore, adolescent drivers are three times more likely to be involved in alcohol-related accidents than adults, with the highest risk among newly licensed drivers [2–4]. Preventative measures against risky driving are particularly pertinent for populations with a high rate of vehicle-involved fatalities [2] and those at increased risk for substance use and the accompanying adverse outcomes [5, 6].

Specifically, driving under the influence of cannabis (DUIC) among young people is a growing public health concern. Although national rates of cannabis-related accidents among young people remain uncertain due to lack of research, the proportion of cannabis-related accidents and fatalities among all drivers has increased since the early 2000s, now accounting for 21% of accidents [7]. More than a quarter of adolescents of driving age report lifetime cannabis consumption, and 12% report daily or regular use, a significant increase from 3% in prior years [8]. Predictors of DUIC include a younger age of first use, higher frequency of consumption of cannabis, consistent heavy drinking, and a history of driving under the influence of alcohol [9]. Those who drive under the influence of cannabis are also at an increased risk of developing cannabis use disorder [10].

Adolescents increasingly view cannabis with more permissive attitudes, which affects rates of impaired driving. As of 2022, only 28% of high school seniors in the U.S. considered regular consumption of cannabis as a “great risk”; a decline from 40% in 2013 and 79% in 1991 [11], highlighting the steady decline in perceived risk of marijuana use over time [12]. This is concerning as a lower perceived risk of cannabis is associated with a greater likelihood of driving under the influence of cannabis [10].

Declines in the perceived risk of cannabis use also affect the prevalence of simultaneous use, defined as consuming alcohol and cannabis within two hours of each other. About 21% of U.S. high school students reported simultaneous use in the past year [13]. This is another concerning trend as simultaneous use is associated with an increased likelihood of driving under the influence or riding with a driver under the influence [14–16]. For example, between 2016 and 2019, 42% of drivers with past-year alcohol and cannabis use reported driving under the influence (8% alcohol-only, 20% cannabis-only, 14% alcohol/cannabis) [17]. Simultaneous use of cannabis and alcohol is also associated with a greater risk of experiencing cognitive impairment, delays in decision-making, and slower reaction times than driving under the influence of either substance alone [18, 19].

Focusing prevention efforts in driver education programs is one way to prevent and reduce driving under the influence and associated substance use-related consequences among adolescents. Given that adolescents typically initiate alcohol and cannabis use around 16 years old, the same age when most learn to drive (16–18 years old) [20, 21], leveraging messaging in driving schools and other driver educational platforms provides an opportunity to disseminate preventative interventions efficiently and effectively. Past interventions focusing on preventing risky substance use in 10th −12th graders demonstrate success in reducing alcohol consumption, cannabis use, and intentions to use in the future [22–24]. These interventions include web based interventions (WBIs) delivered in schools [25–27], primary care [28], and self-directed settings without an instructor [29–32]. However, few studies have evaluated delivering interventions that address the use of both alcohol and cannabis in the context of driving school to prevent risky driving behaviors [33].

This study builds upon an existing brief, single-session intervention in primary care called CHAT [34], which targets alcohol and cannabis initiation, use, and impaired driving. In two trials, CHAT demonstrated reductions in cannabis use and intention to use among adolescents in the short term [35] and over one year [28, 36]. We adapted CHAT for online delivery and focused its content on addressing driving under the influence of both alcohol and cannabis. This study evaluates the feasibility and acceptability of this web-based online intervention (webCHAT) among adolescents in driver education.

## Methods

### Sample and recruitment

Participants were 15–17-year-olds (n = 17), who were in the process of getting their driver’s license from two driver education programs in Michigan and Colorado. Students attending these driver education programs were recruited by driving school staff, who sent invites asking to participate in a focus group or an interview. Parents or guardians provided electronic consent and contact information for their adolescents to the research team, and adolescents were then contacted and assented to participate in either the focus group or individual interview.

### Procedures

We conducted two virtual focus groups (n = 6; n = 5), with adolescents enrolled in these two driver education programs to provide feedback on our intervention prototype and share their experiences with driving. Focus groups were audio recorded with a standalone device and then transcribed for coding. This feedback was integrated into our intervention and the program was then user tested by a new sample of adolescents who provided feedback via a 30-minute phone interview. Of those invited to user test (n = 8), all participants completed the brief survey to assess the intervention’s usability, acceptability, and feasibility, and six participants completed individual qualitative interviews. Data from the interviews was de-identified and descriptive interview notes were used for analysis (due to issues with transferring audio from the recording device). Focus group qualitative data was comprised of direct participant quotes, while individual interviews included descriptive notes from participants taken by the interviewers. All procedures were approved by the Stanford Institutional Review Board.

### webCHAT Intervention

The webCHAT intervention was developed utilizing mechanisms from three theories to prevent risk behaviors: (1) expectancy theory to address negative and positive outcomes related to substance use and driving behavior; (2) social learning theory to address peer modeling and normative beliefs; and (3) decision making theory to address decisions regarding engaging in impaired driving and increasing self-efficacy (Osilla et al., 2023). WebCHAT utilizes motivational interviewing principles and presents vignettes about risky driving situations. It also provides personalized feedback throughout the intervention to help adolescents develop strategies for managing high-risk situations. This intervention was adapted for online delivery via computer or smartphone, to be self-guided, and completed at the participant’s own pace.

WebCHAT focuses on three phases of change: (1) assessing motivation to change; (2) enhancing motivation; and (3) planning ahead. The first phase orients participants to the intervention and how information learned in the intervention can be used to help prevent driving under the influence of alcohol or cannabis. The webCHAT program also collects data on their reported substance use and attitudes about impaired driving to tailor intervention content to their specific needs. The second phase uses personalized normative feedback to provide education on impaired driving and suggest strategies for decreasing driving consequences based on their perceived risks and norms around substance use and risky driving. Finally, the third phase emphasizes protective behavioral strategies using vignettes that display common, high-risk situations associated with impaired driving. Participants then use these protective strategies to role-play different reactions to these different risky situations and assess their willingness and confidence to try these different strategies. The intervention concludes with the narrator summarizing information learned and strategies selected to help the participant plan for future risky situations.

### Measures

#### Feasibility

Participants responded to focus group and interview questions assessing feasibility; defined as the interventions’ usability or the appropriateness of the modality it was delivered in. Topics included reactions to the online modality, ability to navigate the webCHAT program, program format, and potential suggestions to improve retention and engagement. During focus groups, adolescents were probed: “What are your thoughts about having this program on the computer or on a smartphone?” and “What other influential things would you recommend to help keep teens engaged in the information that we want to share?”. During individual interviews, questions included: “How do you think people will react to getting this information on the computer?” and “If you could make one change to this program, what change would you make?”.

#### Acceptability

Participants also evaluated the acceptability of webCHAT; defined as the relatability and accessibility of intervention content, appropriateness of the format for delivering this information, and whether it met their needs. Key topics included an overall impression of intervention content, comparisons to past education or interventions received, feedback on specific sections, potential impacts of the intervention, and if they would recommend the intervention to a friend. Focus group questions included items such as: “If we wanted to share this information about the effects of cannabis and alcohol on driving with teens, what are some important things that you would recommend?”, “What do you think of the narrator, the graphics, the messaging?”, and “What did you think of that [clip]?”. For participants in the individual interviews, these questions included: “What are your overall impressions of the program?”, “How might this program affect teens?”, “How does this program compare to other information or education you’ve received”, and “Would you recommend this program to a friend?”.

#### Analytic plan

We utilized a qualitative approach to identify themes related to the acceptability and feasibility of webCHAT. Two team members (KN, EU) separately reviewed focus group transcripts and interview notes to identify themes. Coders then determined first-level themes within each category using a grounded theory approach [37] and developed a codebook describing each theme, along with inclusion/exclusion criteria, and excerpts from the interviews. Coders used the analysis software Dedoose, a cloud-based software platform that facilitates collaborative management, analysis, and interpretation of qualitative data [38] to complete their ratings, coding independently and then reviewing together. Discrepancies between codes were discussed, and when appropriate, modifications were made to themes and sub-themes, until there was agreement between both coders. Coders were then assessed for level of agreement, which was determined by a Kappa ≥ 0.80. SPSS was used to describe the frequencies of the quantitative data collected during the post-satisfaction surveys.

## Results

### Participant characteristics

Demographic data was not collected from participants to protect anonymity and confidentiality and due to the small sample size. All focus group participants were between the ages of 15 and 17. Interview participants (n = 6) reported a mean age of 15.7 years. Of the six interview participants, a majority were White (67%) and female (67%), with 50% reporting lifetime use of alcohol or cannabis.

### First and Second-Level Themes

Participant feedback was grouped into two outcomes (feasibility and acceptability). Regarding feasibility, participants in the focus groups and interviews commented on the usability or ease of use of the intervention, which was categorized as a theme. Regarding acceptability, comments from focus groups and interviews varied slightly (see Tables 1 and 2). Among focus group participants, sub-themes were grouped under larger themes of study recommendations and their perceptions/experiences. Among interview participants, sub-themes were grouped within larger themes of webCHAT program impressions, program impacts, and perceptions/experiences from the interviews. Coded data from both focus groups and interviews had strong inter-rater reliability (k = 0.99, k = 0.95 respectively).

### Focus Groups

#### Feasibility Usability

##### Online Format.

Focus group participants found the online modality convenient, accessible, less stressful, and easier to voice their experiences and perspectives than in-person settings. One participant appreciated the lack of logistical barriers with the online delivery, stating, “You can just do it without worrying about, oh, can I do this? Is there space? So, you don’t have to stress about it.” The online format was also viewed as more confidential than other settings, with one participant stating, “They can do it without anyone knowing their identity, so that’s always a plus with anything.” None of the participants reported barriers to completion.

##### Program Format.

Focus group participants reported that the program format was appropriate. They enjoyed the informal nature of the program and reported that the narrator had a “friendly tone” and that it felt as if you were “talking to a classmate.” Multiple participants also noted that having a “teen” narrator made them want to listen more, the content seemed more relatable, and they appreciated having options presented to them to think about. They also reported that video clips were an appropriate length and that the graphics were engaging. Participants recommended including bullet points or a summary of the main takeaways throughout the program. Table 3 provides specific focus group participant quotes regarding the feasibility of webCHAT related to online modality and program format.

### Acceptability

#### Study Recommendations

##### Content Enhancements.

Focus group participants recommended: (1) providing anecdotal evidence, such as first-hand stories of those impacted by impaired driving, (2) introducing more interactive components to increase engagement, and (3) providing strategies for talking to peers about risky behavior. Participants also shared that including information on the physiological and cognitive effects of alcohol and cannabis on your body and the legal ramifications of driving under the influence would be helpful. Participants also suggested keeping the intervention as brief as possible.

##### Strategies to Curb Risky Driving.

Focus group participants also discussed potential strategies and alternatives to impaired driving. They emphasized that understanding the potential consequences of driving under the influence is essential in reducing this behavior. Participants also shared the importance of reaching out to an adult or role model and the necessity of designating a driver, planning transportation, and having a backup plan to prevent risky driving. Regarding DUI prevention, participants recommended “knowing the people you’re with,” utilizing ride-share services (i.e., Uber), and identifying a backup plan (i.e., calling your parents or a sibling for a ride). When asked about how they would respond if they were a passenger with a driver under the influence, they recommended “staying calm,” “helping the driver to stay calm too,” and persuading “them to pull over.” Table 4 provides additional quotes from focus group participants on ways to enhance the content and strategies discussed in the intervention.

##### Perceptions and Experiences.

Focus group participants also provided feedback on their experiences, perceptions regarding the prevalence of use, and their beliefs about driving under the influence to inform the development of tailored content for this population.

##### Previous Education on and Experience with Risky Driving.

Participants reported that their previous driver education primarily consisted of “don’t do it” messages and activities involving “drunk goggles” to discourage such behavior. Regarding previous experiences, participants expressed di culties when faced with situations as a passenger where the driver was under the influence, particularly when the driver was an “older family member who they are supposed to respect” or when “they are the only one who perceives the situation as wrong or risky.”

##### Availability and Use of Alcohol and Cannabis.

When focus group participants were asked to compare perceptions of the availability and use of alcohol and cannabis, the majority stated that cannabis was more prevalent and available. Participants also shared that the legalization of cannabis increased the normalization of using the substance. Participants felt cannabis was more prevalent because it was easier to access and easier to hide, and that negative consequences associated with use are not as well-known as those for alcohol.

##### Beliefs About Driving Under the Influence.

Focus group participants reported mixed views regarding the prevalence of driving under the influence of either cannabis or alcohol. Some stated it was less common now due to ride-share services and an increase in adolescents holding their friends accountable, whereas others shared that it occurs frequently, especially when the police arrive at parties, which causes people to leave in a rush, or when going to “afterparties”, or events that occur after a main party or event. Although perceptions on DUI varied, all focus group participants (n = 11) noted that DUI occurred regularly, with one stating that “a much [larger] group than older generations” drive under the influence. Participants also reported that adolescents perceive DUIC less seriously than alcohol because they feel more in control of their bodies, and many adolescents are either unaware or do not believe that cannabis impairs their executive functioning. Participants shared that they and their peers experience more paranoia about driving under the influence of alcohol, leading adolescents to be more willing to use cannabis and drive as an alternative. Table 5 provides additional focus group quotes regarding adolescent perceptions, experiences, and beliefs about cannabis and alcohol.

### Translating Focus Group Feedback

Feedback and recommendations from focus group participants regarding the feasibility and acceptability of webCHAT were incorporated into the next iteration of the program. For feasibility, participants recommended utilizing bullet points after each section to help with summarizing the main takeaways; thus, we developed summary slides throughout the program. Participants also shared recommendations to improve the acceptability of webCHAT content by including first-hand vignettes of those impacted by impaired driving, with interactive components, and providing clear strategies on how to communicate with your peers about risky behaviors. Therefore, we incorporated videos of adolescents describing common situations and experiences and consequences that can occur, the impacts of risky driving, interactive questionnaires throughout the program to increase engagement, and we provided additional communication strategies for navigating difficult peer conversations.

Focus group participants shared specific examples and experiences they have had with risky driving and substance use, to help create tailored content in the program (e.g., what to say at a party or game where people are using and then need to drive home). Participants were curious about the risks of cannabis use and alcohol, which led us to develop more focused education around simultaneous use. Focus group participants also shared important considerations and strategies that they currently use to reduce risky or impaired driving, such as identifying backup plans and utilizing alternative transportation, which we integrated into the strategy section of the program.

### Individual Interviews

#### Feasibility Usability

##### Online Format.

Interview participants who user-tested webCHAT largely expressed positive views (67%) regarding the online intervention modality. They found the online approach feasible, indicating that it would be easier to do the intervention on computers because it facilitates more honest reporting, facilitates an increased sense of privacy, and feels more informal than in person. However, one participant preferred a classroom setting and another suggested that some adolescents might opt out of the intervention without someone holding them accountable to complete it.

##### Program Format.

Participants responded favorably to content delivery, sharing that the scenarios are more effective than scare tactics. However, they recommended making skits less formal and adding more “hands-on” or engaging experiences to help reiterate the information shared. Some participants also suggested extending the program beyond a single session, as repeated exposure may be even more impactful. Table 6 provides additional notes on the usability feedback on the program for participants.

### Acceptability

#### Program Impressions

##### Initial Reactions.

All interview participants said that they would recommend the program to a friend (n = 6). One participant noted that the intervention was useful, but they were already familiar with the information, and another noted that it would be useful to have a space afterward to discuss the content. Interview participants also stated that information regarding cannabis was not commonly included in their previous education and was appreciated. Largely, participants reported that webCHAT included an abundance of useful information and that the program was insightful and realistic.

##### Program Content.

Most interview participants responded positively to intervention content, with five participants (83%) reporting overall positive perceptions of webCHAT. They found the intervention’s organization and engaging format acceptable. Specifically, they appreciated the inclusion of videos that shared non-judgmental perspectives, prevention strategies, and real statistics on the perceptions and behaviors of their peers. Participants also found the mixture of survey questions and response comparisons enjoyable and appreciated that the videos were short and digestible. Participants also reported appreciating the testimonials about impaired driving and demonstrations on how to safely remove yourself from situations with impaired drivers. They found webCHAT content to be more helpful than other interventions because it provided actionable strategies, relatable content and scenarios with adolescent actors, avoided scare tactics, focused on friends and peers, and was more engaging, clear, data-focused, and less fearmongering than alternatives. Table 7 provides additional notes regarding the program impressions for user testing interview participants.

#### Program Impacts

##### Changes to Substance Use Behaviors and Driving.

Two interview participants (33%) stated that they believed the intervention could help adolescents make changes in their substance use behaviors. They believed it would help adolescents reconsider driving under the influence and enable them to make more informed decisions if/when they are under the influence of alcohol or cannabis. They also shared that it would help adolescents establish preventative plans in high-risk situations. However, one participant noted that the program might not be impactful because they may forget the information, and another stated it was “useful and educational, but not sure how impactful”.

##### Learning/New Insights.

During the interviews, five (83%) participants reported that webCHAT was beneficial and helped them learn new things. Participants stated that they gained new perspectives and learned about risky behaviors, impaired driving, substance use, DUI statistics, and the effects of cannabis on driving. One participant mentioned having seen similar videos in the past. Table 8 provides additional notes regarding program impact feedback from user testing interview participants.

#### Perceptions & Experiences

##### Perceived Rates of Substance Use in Peers.

When asked about the prevalence of peer substance use, four (67%) interview participants reported that drug and alcohol use was prevalent in both public and private schools, with some using weekly, even if their peers don’t use these substances. One participant also noted that substance use among adolescents mostly occurs at parties.

##### Availability and Use of Alcohol and Cannabis.

Participants from interviews echoed similar substance use perceptions as focus group participants. They noted that adolescents often combine alcohol and cannabis, with only some using one at a time. Participants also highlighted that adolescents perceive cannabis as a safer option to use while driving compared to alcohol. They stated that this was a widespread perception amongst peers their age and that DUIC is not as overtly dangerous as driving under the influence of alcohol (DUIA). Table 9 provides additional notes regarding adolescent perceptions and experiences in user testing interviews.

### User Satisfaction Surveys

Eight participants from the user testing completed the post-intervention user satisfaction surveys. All participants (100%) rated the quality of the webCHAT as good or excellent. Six participants (88%) agreed that they would recommend webCHAT to a friend and that they could understand and were satisfied with the information presented. Six participants (88%) also reported that they learned how to prevent impaired driving, could use the information when they drive, and that they learned skills to better manage their lives. Five participants (75%) agreed that participating in webCHAT could contribute to leading a healthier life (Table 10).

## Discussion

The current study provides preliminary data that an online, brief intervention to prevent risky driving behaviors among adolescents in driving school is both feasible and acceptable. Incorporating participant feedback before piloting an intervention can enhance the intervention’s quality and acceptability, especially when targeting hard-to-reach groups such as adolescents. During both focus groups and interviews, the general impression of the program was positive, indicating high feasibility for this web-based format. To increase the intervention’s acceptability, focus group participants suggested including risky driving scenarios and strategies throughout the intervention. Participants reported personal views and experiences with substance use and risky driving to inform additional intervention content revisions. Through user testing interviews, participants shared that they were satisfied with the program, reporting high ratings on both the quality of webCHAT and their willingness to recommend webCHAT to a friend. However, participants also shared concerns that an online, self-guided format may contribute to a lack of accountability and motivation to complete the program. Despite these reported barriers in feasibility, all user testing participants completed the entire program. Overall, findings across focus groups, interviews, and satisfaction surveys demonstrate that the program’s scope, content, and format were highly acceptable.

Feedback from focus groups and interviews indicated a need for alternative educational approaches regarding substance use, criticizing the ineffectiveness of “scare tactics” that are often used. Participants also felt that other impaired driving interventions offer few statistics, rarely contain relatable content, and lack advice tailored to adolescents, including education on cannabis. As a result, webCHAT’s use of real data and inclusion of strategies to reduce impaired driving was seen as a more suitable alternative, improving the program’s acceptability. This aligns with past research emphasizing the importance of making adolescents feel seen and respected when developing similar interventions [39]. Our findings suggest that the online modality of webCHAT was well-liked by participants, consistent with other online interventions preventing substance use in this population [22]. Future interventions should consider using relatable narrators, testimonials, ample statistics, and frequent summaries in their content, and also seek adolescent input to maximize information uptake and optimize outcomes.

This study has several limitations. We had a small sample size and lack of racial and ethnic diversity, which limited applicability to a broader population. We also only recruited from two driver education schools in suburban Michigan and Colorado, which limits our knowledge of the intervention’s feasibility and generalizability in other regions. Furthermore, participants may have unequal familiarity and access to technology, which could further limit the generalizability of results and may have caused bias, whereby participants with access may be the only ones to participate. Our qualitative interview data is also limited due to technical difficulties with the recorder, meaning themes were identified through the use of facilitator notes from the interview. Finally, we recognize selection bias in recruitment as a limitation, as adolescents less likely to engage in these behaviors may have been more likely to participate. Future studies should aim to recruit more diverse samples to provide a better understanding of how the acceptability and feasibility of such an intervention may vary based on participant characteristics or other social determinants. Feedback from this qualitative study will guide the next phase: a pilot randomized controlled trial (RCT) that will assess the effectiveness of webCHAT combined with usual driver education versus usual driver education alone [40]. Although many substance use interventions are available for young people in school and primary care settings, webCHAT offers a unique program to provide preventative messaging at an opportune time when adolescents are learning to drive

## Conclusions

This preliminary data suggests that webCHAT is a feasible and acceptable intervention to address cannabis and alcohol use for adolescents learning to drive. This study illuminates the importance of soliciting adolescent perspectives when developing interventions, and especially those targeting cannabis use. This intervention could help address the growing public health concerns of impaired driving and DUIC among adolescents.

## Figures and Tables

**Table 1:** Themes and Sub-themes from focus groups.

Outcome	Theme	Sub-Themes
Feasibility	Usability	Online format
		Program format
Acceptability	Study Recommendations	Content enhancements
		Strategies to curb risky driving behaviors
	Perceptions and Experiences	Previous education on and experience with risky driving
		Availability and use of alcohol and cannabis
		Beliefs about driving under the influence

**Table 2:** Themes and Sub-themes from user testing interviews.

Outcome	Theme	Sub-Themes
Feasibility	Usability	Online format
		Program format
Acceptability	Program Impressions	Initial reactions
		Program content
	Program Impacts	Changes to substance use behaviors and driving
		Learning/new insights
	Perceptions and Experiences	Perceived rates of substance use in peers
		Availability and use of alcohol and cannabis

**Table 3:** Illustrative quotes for the Usability theme from focus groups.

Sub-theme	Participant Quotes
Online modality	“It’s more convenient. That’s for sure”
	“They can do it without anyone knowing their identity, so that’s always a plus with anything.”
	“I think it makes it more accessible for a lot of people which is good”
	“And that way you don’t have to find the specific place to go and do the class. And you don’t have to sign up and there be a certain amount of people that can go”
	“I also think it makes it easier for teens and parents to share it. Because it’s just a link you can easily send to someone and be like, hey, have your kids check this out.”
Program format	“I liked how her [narrator] tone was very friendly like she just sounded friendly and not very harsh, I guess. I don’t know.”
	“The narrator being what I think is a teen makes it—like, teens want to listen more than if it was an adult or teacher who tells us this stuff anyway.”
	“It was kind of interesting to see what she had to say. So yeah, if I was watching that video as a student in that class, I would pay more attention to see what teens have to offer as their opinion, after seeing her speak.”
	“When you can relate to someone and things are relatable, it impacts you more than— if it’s positive things or negative things.”
	“I also think that the fact that the clip wasn’t too long was good.”
	“I also really liked the line where it was like, “See if their ideas match yours,” because every single teen has a different perspective on driving under the influence. And so, I think that giving options really is effective.”
	“I think it’s nice that everything the narrator said wasn’t typed out. Like it’s just little pieces of it. So, it’s easier to be engaged with smaller pieces.”
	“I think it’s [the graphic] also good because it keeps the teens engaged. Because there’s a lot to look at, and it’s not super bland and boring and just here’s a text box, read it.”

**Table 4:** Illustrative quotes for the Study Recommendations theme from focus groups.

Sub-theme	Participant Quotes
Content enhancements	“… having a teen who’s experienced a situation with drunk driving or who knew someone … who maybe passed away … and feels super passionate about it come in.”
	“… how to talk to your peers about it and talk to a peer who is going to drive under the influence.”
	“… the effects that it can have on your body because I feel like people don’t talk about that at all, in terms of while driving.”
	“Maybe a conversation starter list of have your parent come over and watch this segment with you or something like that.”
	“That it doesn’t matter how much food you eat, it doesn’t change your blood alcohol content.”
	“Yeah, or teens should be better informed about drinking and driving or using drugs while driving. I think it should be more impressed on teens on what could happen, or what will happen, or how this could ruin your life and it’s not worth it.”
	“It has to be interactive, and it just has to be different”
	“Maybe like have it short, like 15—like half-an-hour long because then they’ll probably stop listening after that. So just keep it concise but multiple sessions around that time.”
Strategies to curb risky driving	“Having a designated driver and then if something happens with them and they don’t end up going or if they end up being pressured into drinking, then having the ability to have a friend’s house close by that you can stay at without having to drive to.”
	“I think just think about your decision before you enter any vehicle while under the influence or while somebody else is.’
	“You can Uber there instead of driving there.”
	“I think the big part is you have to plan in advance.”
	“If you’re not getting in the car, trying to grab their keys from them [driver under the influence] or something like that.”

**Table 5:** Illustrative quotes for the Perceptions and Experiences theme from focus groups.

Sub-theme	Participant Quotes
Previous education on and experience with risky driving	“People don’t go into a lot of depth about explaining it. They just say don’t do it.
	“I think especially with driver’s ed, they have to put the drunk goggles on and walk around and try to do tasks.”
	“And they just told us not to do it, and they had us do fun activities. And then after each activity they just said, don’t do it.”
	“I honestly don’t remember being talked to about driving under the influence of marijuana.”
	“Sometimes it’s very difficult, though, especially when someone’s older than you.”
	“Or if you’re in a car and the car is full of people and no one thinks that it’s wrong except for you, it’s kind of hard to make everyone think it’s wrong.”
Availability and use of alcohol and cannabis	“I think it’s [cannabis] a lot more common than alcohol”
	“I think it’s [cannabis] sort of normalized a lot. I think it’s a lot more normalized in our generation than before, because of legalization.”
	“It’s just easier to access it [cannabis].”
	“I would say it’s [cannabis] more [common] than drinking alcohol in the State of Colorado, if you ask me. I would say it’s more than drinking alcohol because teens just seem to do it more.”
	“People that are already vaping, they get bored eventually from whatever, and then they start smoking marijuana, too, or they mix both of them.”
	“I would say it’s easier to hide smoking than drinking.”
Beliefs about driving under the influence	“I think that it’s not as common as it used to be… there’s a lot of fear instilled in this generation about drunk driving.”
	“I think it happens a lot of the times when cops show up because people just freak out and they have to just leave. So then there’s a lot of drunk driving there.”
	“I also think even people who say they’re going to be a designated driver, they get peer pressured into drinking, too. And you don’t have your designated driver.”
	“At least in my city, I know that a lot of my friends and people I know, in general, if we know someone’s drunk, we don’t let them drive… So it’s not that frequent, for me at least.”
	“Football games and dances, school dances it happens all the time.”
	“That’s where the main thing happens because people get drunk or high. And then they go to a dance, and then they drive to the afterparties.”
	“I think it’s more than you guys know. I think a lot of teens do it more than you guys think drink or use drugs while driving”
	“I feel like teenagers always think, like, they’re kind of invincible in a way. They think nothing bad is going to happen, so they just take the chance and take the risk. So I think that’s why so many teens drink while under the influence or drive when under the influence.”

**Table 6:** Illustrative notes for the Usability theme from user testing interviews.

Sub-theme	Participant Notes
Online format	It’s easier to do on the computer, you can be more honest.
	Most people would be more comfortable doing it live in class and if mandatory, it would be better.
	Easy to use, didn’t have troubles.
	Thought the computer was effective because it felt casual and less intense.
	Thinks a majority of teenagers would opt out because there isn’t someone “motivating” them to complete.
Program format impressions	Scenarios are definitely more effective, scare-tactics are corny.
	Would like a real-life example of what it’s like driving impaired, what that looks like, understand what that would be like.
	More interactivity, engaging hands-on experiences to help drive home the points
	I want to see more of the data.
	Teens need to understand that this is more pervasive than they think.
	Adding testimonials from real people who have been impacted by impaired driving makes it feels “real”.
	Make it a longer-term program with repeated exposure.
	More real demonstrations of how to get out of unsafe situations.
	Better if high school was able to present it, driving school isn’t as effective and there are a lot of different programs.

**Table 7:** Illustrative notes for the Program Impressions theme from user testing interviews.

Sub-theme	Participant Notes
Initial Reactions	Felt it was very in line with what she expected, felt nothing was out of the norm. The marijuana portion was new and appreciated.
	Really liked the tools he was given to keep himself out of bad/unsafe situations.
	Would recommend this to a friend, as there was lots of useful information.
	Liked the informality of it and that she could do it on her own but thinks a space to have conversation is useful.
	It was useful, but he knew all the info.
	It was interesting and had a lot of questions that he hadn’t personally experienced, so it was insightful.
	Feels like other programs show the “worst of the worst” and he liked that this was more realistic.
Program Content Perceptions	Felt the other programs don’t give him actionable strategies and he liked that we do provide scenarios.
	Other programs need to be more relatable like how information is presented in WebCHAT, scare tactics are not as effective.
	Continue to share a non-judgmental perspective.
	More relatable as these situations “actually happened”.
	Loved the data, helped “open her eyes” to the prevalence.
	Liked the predictive statistics and then showing the real statistics.
	For the multiple choice questions/survey questions, they really liked being able to compare responses after watching videos.
	Lots of videos were used but liked that they were short and easily understandable
	More clarity, more engaging; more data-focused, less fearmongering [than other interventions].

**Table 8:** Illustrative notes for the Program Impacts theme from user testing interviews.

Sub-theme	Participant Notes
Changes to substance use behaviors	Might help them to make more informed decisions, thinking twice before doing it.
	Less likely that teens will engage in unsafe behaviors, and more likely to have preventative plans in place.
	Feels that she has forgotten a lot of the info, so it’s difficult to carry with you long-term and may not be as impactful to use.
Learning/new insights	Learned about the perspective of what other people experience, how they view risky behaviors, and what would impair you.
	You need to have a trusted adult that you’re comfortable with, so you can leave a party or bad situation.
	How low the statistics actually were for her peer group in terms of these behaviors
	Emphasis on marijuana and how it impacts driving - was under the impression it wasn’t as dangerous.
	[Learned] not to smoke and drive
	Learned about situations he hasn’t yet been in but felt it was helpful.

**Table 9:** Illustrative notes for the Perceptions and Experiences theme from user testing interviews.

Sub-theme	Participant Notes
Perceived rates of substance use in peers	In public and private schools, felt drug and alcohol abuse was prevalent and that teens often combined frequently.
	A lot of the population at his school uses both but not his friend group.
	Feels like there are a handful of people who engage weekly, and that most drive afterward.
	People use them at parties, using them together often, and older siblings as a bad influence.
Availability and use of alcohol and cannabis	Alcohol is worse [than cannabis].
	One or the other [alcohol or cannabis], not common to do both [alcohol and cannabis]
	You drink first and then smoke; you don’t smoke and then drink.
	Preference for one over the other usually, it’s not as common to combine both, “cross-faded” is a horrible experience and not to do it.
	People view marijuana differently than alcohol and view it as safer.
	People believe that marijuana actually makes you more attentive and this is a common misconception in her age group.
	More people drink but recognize that it’s not a safe behavior to do while driving, whereas with marijuana it feels like more of a gray area and less “overtly” bad.
	More fear around “what is in it” when it comes to marijuana, especially edibles - feels less controlled so alcohol may feel more natural.

**Table 10:** User satisfaction survey results (n = 8).

Survey Question	Agreed or Strongly Agreed (%)
I would recommend it to a friend	88
I could understand the information	88
I am satisfied with the information	88
The different videos were helpful	88
I learned more about how to prevent impaired driving	88
I could use information from it when I drive	88
The things I did will help me drive safely	88
It helped me learn skills to better manage my life	88
Participating can help me lead a healthier life	75

## Data Availability

The datasets during and/or analyzed during the current study available from the corresponding author on reasonable request.

## Abbreviations

DUIC: Driving under the influence of cannabis
DUIA: Driving under the influence of alcohol

## References

[1] National Highway Traffic Safety Administration. How alcohol affects driving ability. 2020. Available: https://www.nhtsa.gov/risky-driving/drunk-driving. Accessed 13 November 2023.

[2] Centers for Disease Control and Prevention. Teen Drivers and Passengers: Get the Facts. 2022. Available: https://www.cdc.gov/transportationsafety/teen_drivers/teendrivers_factsheet.html. Accessed 13 November 2023.

[3] Lombardi DA, Horrey WJ, Courtney TK. Age-related differences in fatal intersection crashes in the United States. Accid Anal Prev. 2017;99: 20–29. doi:10.1016/j.aap.2016.10.030 27855312

[4] Missikpode C, Peek-Asa C, McGehee DV, Torner J, Wakeland W, Wallace R. Teen driver system modeling: a tool for policy analysis. Inj Epidemiol. 2018;5: 34. doi:10.1186/s40621-018-0164-9 30221317 PMC6139293

[5] Gray KM, Squeglia LM. Research Review: What have we learned about adolescent substance use? J Child Psychol Psychiatry. 2018;59: 618–627. doi:10.1111/jcpp.12783 28714184 PMC5771977

[6] Jordan CJ, Andersen SL. Sensitive periods of substance abuse: Early risk for the transition to dependence. Dev Cogn Neurosci. 2017;25: 29–44. doi:10.1016/j.dcn.2016.10.004 27840157 PMC5410194

[7] Lira MC, Heeren TC, Buczek M, Blanchette JG, Smart R, Pacula RL, Trends in Cannabis Involvement and Risk of Alcohol Involvement in Motor Vehicle Crash Fatalities in the United States, 2000‒2018. Am J Public Health. 2021;111: 1976–1985. doi:10.2105/AJPH.2021.306466 34709858 PMC8630490

[8] Johnston LD, Miech RA, O’Malley PM, Bachman JG, Schulenberg JE, Patrick ME. Monitoring the Future national survey results on drug use 1975–2021: Overview, key findings on adolescent drug use. Ann Arbor: Institute for Social Research, University of Michigan.; 2022.

[9] Sterzer FR, Caird JK, Simmons S, Bourdage JS. A scoping review of predictors of driving under the influence of cannabis (DUIC) in young drivers. Transp Res Part F Traffic Psychol Behav. 2022;88: 168–183. doi:10.1016/j.trf.2022.05.014

[10] Cantor N, Kingsbury M, Hamilton HA, Wild TC, Owusu-Bempah A, Colman I. Correlates of driving after cannabis use in high school students. Prev Med. 2021;150: 106667. doi:10.1016/j.ypmed.2021.106667 34081937

[11] Miech RA, Johnston LD, Patrick ME, O’Malley PM, Brachman JG, Schulenberg JE. Monitoring the Future national survey results on drug use, 1975–2022: Secondary school students. Ann Arbor, MI: Institute for Social Research, University of Michigan; 2023. Available: https://monitoringthefuture.org/results/publications/monographs/. Accessed 13 November 2023.

[12] Dunn MS, Klump MA. Perceived Risk of Weekly Marijuana Use Among Students Participating in the National Survey on Drug Use and Health. J Drug Issues. 2023; 00220426231207779. doi:10.1177/00220426231207779

[13] Patrick ME, Kloska DD, Terry-McElrath YM, Lee CM, O’Malley PM, Johnston LD. Patterns of simultaneous and concurrent alcohol and marijuana use among adolescents. Am J Drug Alcohol Abuse. 2018;44: 441–451. doi:10.1080/00952990.2017.1402335 29261344 PMC6027645

[14] Lipperman-Kreda S, Gruenewald PJ, Grube JW, Bersamin M. Adolescents, alcohol, and marijuana: Context characteristics and problems associated with simultaneous use. Drug Alcohol Depend. 2017;179: 55–60. doi:10.1016/j.drugalcdep.2017.06.023 28755540 PMC5599344

[15] Paschall MJ, García-Ramírez G, Grube JW. Recreational Marijuana Legalization and Co-use With Alcohol Among Adolescents. Am J Prev Med. 2022;62: 57–64. doi:10.1016/j.amepre.2021.06.003 34426059 PMC8688237

[16] Patrick ME, Graupensperger S, Dworkin ER, Duckworth JC, Abdallah DA, Lee CM. Intoxicated driving and riding with impaired drivers: Comparing days with alcohol, marijuana, and simultaneous use. Drug Alcohol Depend. 2021;225: 108753. doi:10.1016/j.drugalcdep.2021.108753 34058538 PMC8282726

[17] Gonçalves PD, Gutkind S, Segura LE, Castaldelli-Maia JM, Martins SS, Mauro PM. Simultaneous Alcohol/Cannabis Use and Driving Under the Influence in the U.S. Am J Prev Med. 2022;62: 661–669. doi:10.1016/j.amepre.2021.11.009 35459450 PMC9038028

[18] Duckworth JC, Lee CM. Associations among simultaneous and co-occurring use of alcohol and marijuana, risky driving, and perceived risk. Addict Behav. 2019;96: 39–42. doi:10.1016/j.addbeh.2019.03.019 31030178 PMC6579674

[19] Lee CM, Cadigan JM, Patrick ME. Differences in reporting of perceived acute effects of alcohol use, marijuana use, and simultaneous alcohol and marijuana use. Drug Alcohol Depend. 2017;180: 391–394. doi:10.1016/j.drugalcdep.2017.08.029 28972908 PMC5690541

[20] Tefft BC, Foss RD. Prevalence and Timing of Driver Licensing Among Young Adults, United States, 2019. Washington, D.C: AAA Foundation for Traffic Safety; 2019.

[21] National Institute on Alcohol Abuse and Alcoholism. Get the Facts About Underage Drinking. U.S. Department of Health and Human Services; 2023.

[22] Champion KE, Newton NC, Barrett EL, Teesson M. A systematic review of school-based alcohol and other drug prevention programs facilitated by computers or the internet. Drug Alcohol Rev. 2013;32: 115–123. doi:10.1111/j.1465-3362.2012.00517.x 23039085

[23] Hennessy EA, Tanner-Smith EE. Effectiveness of Brief School-Based Interventions for Adolescents: A Meta-analysis of Alcohol Use Prevention Programs. Prev Sci. 2015;16: 463–474. doi:10.1007/s11121-014-0512-0 25294110 PMC4636019

[24] Lemstra M, Bennett N, Nannapaneni U, Neudorf C, Warren L, Kershaw T, A systematic review of school-based marijuana and alcohol prevention programs targeting adolescents aged 10–15. Addict Res Theory. 2010;18: 84–96. doi:10.3109/16066350802673224

[25] Ariza C, Pérez A, Sánchez-Martínez F, Diéguez M, Espelt A, Pasarín MI, Evaluation of the effectiveness of a school-based cannabis prevention program. Drug Alcohol Depend. 2013;132: 257–264. doi:10.1016/j.drugalcdep.2013.02.012 23490453

[26] Champion KE, Newton NC, Stapinski L, Slade T, Barrett EL, Teesson M. A cross-validation trial of an Internet-based prevention program for alcohol and cannabis: Preliminary results from a cluster randomised controlled trial. Aust N Z J Psychiatry. 2016;50: 64–73. doi:10.1177/0004867415577435 25801662

[27] Newton NC, Andrews G, Champion KE, Teesson M. Universal Internet-based prevention for alcohol and cannabis use reduces truancy, psychological distress and moral disengagement: A cluster randomised controlled trial. Prev Med. 2014;65: 109–115. doi:10.1016/j.ypmed.2014.05.003 24823906

[28] D’Amico EJ, Parast L, Shadel WG, Meredith LS, Seelam R, Stein BD. Brief motivational interviewing intervention to reduce alcohol and marijuana use for at-risk adolescents in primary care. J Consult Clin Psychol. 2018;86: 775–786. doi:10.1037/ccp0000332 30138016 PMC6110093

[29] Budney AJ, Stanger C, Tilford JM, Scherer EB, Brown PC, Li Z, Computer-assisted behavioral therapy and contingency management for cannabis use disorder. Psychol Addict Behav. 2015;29: 501–511. doi:10.1037/adb0000078 25938629 PMC4586287

[30] Copeland J, Rooke S, Rodriquez D, Norberg MM, Gibson L. Comparison of brief versus extended personalised feedback in an online intervention for cannabis users: Short-term findings of a randomised trial. J Subst Abuse Treat. 2017;76: 43–48. doi:10.1016/j.jsat.2017.01.009 28162849

[31] Rooke S, Copeland J, Norberg M, Hine D, McCambridge J. Effectiveness of a Self-Guided Web-Based Cannabis Treatment Program: Randomized Controlled Trial. J Med Internet Res. 2013;15: e26. doi:10.2196/jmir.2256 23470329 PMC3636012

[32] Tossmann DrH-P, Jonas B, Tensil M-D, Lang P, Strüber E. A Controlled Trial of an Internet-Based Intervention Program for Cannabis Users. Cyberpsychology Behav Soc Netw. 2011;14: 673–679. doi:10.1089/cyber.2010.0506 21651419

[33] Razaghizad A, Windle SB, Gore G, Benedetti A, Ells C, Grad R, Interventions to Prevent Drugged Driving: A Systematic Review. Am J Prev Med. 2021;61: 267–280. doi:10.1016/j.amepre.2021.03.012 34099354

[34] Stern SA, Meredith LS, Gholson J, Gore P, D’Amico EJ. Project CHAT: A brief motivational substance abuse intervention for teens in primary care. J Subst Abuse Treat. 2007;32: 153–165. doi:10.1016/j.jsat.2006.07.009 17306724

[35] D’Amico EJ, Miles JNV, Stern SA, Meredith LS. Brief motivational interviewing for teens at risk of substance use consequences: A randomized pilot study in a primary care clinic. J Subst Abuse Treat. 2008;35: 53–61. doi:10.1016/j.jsat.2007.08.008 18037603

[36] D’Amico EJ, Parast L, Osilla KC, Seelam R, Meredith LS, Shadel WG, Understanding Which Teenagers Benefit Most From a Brief Primary Care Substance Use Intervention. Pediatrics. 2019;144: e20183014. doi:10.1542/peds.2018-3014 31296568 PMC6746575

[37] Corbin JM, Strauss AL, Strauss AL. Basics of qualitative research: techniques and procedures for developing grounded theory. 3rd ed. Los Angeles, Calif: Sage Publications, Inc; 2008.

[38] Consultants SR. Dedoose. In (Version Dedoose version 8.2.32) SocioCultural Research Consultants, LLC; 2019. Available: www.dedoose.com

[39] Yeager DS, Dahl RE, Dweck CS. Why Interventions to Influence Adolescent Behavior Often Fail but Could Succeed. Perspect Psychol Sci. 2018;13: 101–122. doi:10.1177/1745691617722620 29232535 PMC5758430

[40] Osilla KC, D’Amico EJ, Smart R, Rodriguez A, Nameth K, Hummer J. Study design to evaluate a web-intervention to prevent alcohol and cannabis-impaired driving and use among adolescents in driver education. Addict Sci Clin Pract. 2023;18: 17. doi:10.1186/s13722-023-00373-2 36964608 PMC10037905

